# 

**Published:** 1899-03-11

**Authors:** 


**Notices of Births, Deaths, and Marriages** are inserted in this column at a charge of 2s. 6d. for an announcement not exceeding four lines.

### NOTICE TO CORRESPONDENTS.

All MS., Letters, Books for Review, and other matters intended for the Editor should be addressed The Editor, "The Hospital," The Hospital Building, 28 & 29, Southampton Street, Strand, W.C.
The Editor cannot undertake to return rejected MS., even when accompanied by stamped directed envelope.

**Notice to Advertisers and Subscribers.**

All Advertisements, Orders for Copies of The Hospital, and Business Communications should be addressed to **THE MANAGER (Not to the Editor),** The Hospital, The Hospital Building, 28 & 29, Southampton Street, Strand, London, W.C.
The Cost of Subscription, post free, is as follows:—Per week, 2½d.; per quarter, 2s. 8d.; per half-year, 5s. 3d.; per annum, 10s. 6d. Foreign:—Per annum, 15s. 2d., payable, in all cases, in advance.

**NOTICE.—Vol. XXIV. of "The Hospital" (April, 1898, to September, 1898), in handsome cloth binding, is now on sale at the Office of this Journal, price 7s. 6d. Cases for binding the Numbers contained in this Volume 1s. 6d. Index to Vol. XXIV., 2½d., post free.**

**The Monthly Part for March is now ready. Price 6d., by post 8d.**

### THE STUDENT OF MEDICINE.

St. Thomas's Hospital, House Appointments.—The following gentlemen have been selected as House Officers from Tuesday, March 7th, 1899:—House Physicians: G. B. Thwaites, L.R.C.P., M.R.C.S. (extension), E. A. Gates, L.R.C.P., M.R.C.S., A. E. Stevens, M.B.Durham, L.R.C.P., M.R.C.S., and H. D. Singer, M.B.Lond., L.R.C.P., M.R.C.S. (extension). Assistant House Physicians: E. H. Ross, L.R.C.P., M.R.C.S., and H. C. Thorp, M.A., M.B., B.C.Camb. House Surgeons: S. O. Bingham, L.R.C.P., M.R.C.S., E. M. Corner, M.A., M.B., B.C.Camb., B.Sc.Lond., L.R.C.P., M.R.C.S., J. A. Barnes, L.R.C.P., M.R.C.S., and J. E. Kilvert, L.R.C.P., M.R.C.S. Assistant House Surgeons; H. J. Phillips, L.R.C.P., M.R.C.S., P. W. G. Sargent, M.A., M.B., B.C.Camb., L.R.C.P., M.R.C.S., S. A. Lucas, L.R.C.P., M.R.C.S., and H. T. D. Acland, L.R.C.P., M.R.C.S. Obstetric House Physicians: (Senior) R. H. Bell, M.A., M.B., B.C.Camb., L.R.C.P., M.R.C.S., and (Junior) S. H. Belfrage, M.B.Lond., L.R.C.P. M.R.C.S. Ophthalmic House Surgeons: (Senior) J. S. Hall, L.R.C.P., M.R.C.S., and (Junior) T. Hoban, L.R.C.P., M.R.C.S. Clinical Assistants in the Special Department for Diseases of the Throat: W. C. Ambrose, B.A.-Camb., L.R.C.P., M.R.C.S. (extension), and E. C. Bourdas, L.R.C.P., M.R.C.S. Clinical Assistants in the Special Department for Diseases of the Skin: H. M. Scaping, B.A.Camb., L.R.C.P., M.R.C.S. (extension), and J. Gaff, L.R.C.P., M.R.C.S. Clinical Assistant in the Special Department for Diseases of the Ear: A. W. Jones, L.R.C.P., M.R.C.S. Clinical Assistants in the Electrical Department: H. N. Goode, L.R.C.P., M.R.C.S. (extension), and A. Bevan, L.R.C.P., M.R.C.S.

